# Flexible Polyurethane Foams Modified with Novel Coconut Monoglycerides-Based Polyester Polyols

**DOI:** 10.1021/acsomega.3c07312

**Published:** 2024-01-15

**Authors:** Christine Joy M. Omisol, Blessy Joy M. Aguinid, Gerson Y. Abilay, Dan Michael Asequia, Tomas Ralph Tomon, Karyl Xyrra Sabulbero, Daisy Jane Erjeno, Carlo Kurt Osorio, Shashwa Usop, Roberto Malaluan, Gerard Dumancas, Eleazer P. Resurreccion, Alona Lubguban, Glenn Apostol, Henry Siy, Arnold C. Alguno, Arnold Lubguban

**Affiliations:** † Center for Sustainable Polymers, MSU-Iligan Institute of Technology, Iligan City 9200, Philippines; ‡ Graduate Program of Materials Science and Engineering, Department of Material Resources Engineering and Technology, MSU-Iligan Institute of Technology, Iligan City 9200, Philippines; § Department of Chemical Engineering and Technology, MSU-Iligan Institute of Technology, Iligan City 9200, Philippines; ∥ Department of Chemistry, The University of Scranton, Scranton, Pennsylvania 18510, United States; ⊥ Montana State University-Northern, Havre, Montana 59501, United States; # Department of Mathematics, Statistics, and Computer Studies, University of the Philippines Rural High School, Paciano Rizal, Bay, Laguna 4033, Philippines; ∇ Chemrez Technologies, Inc., Quezon City 1110, Philippines; ◆ Department of Physics, MSU-Iligan Institute of Technology, Iligan City 9200, Philippines

## Abstract

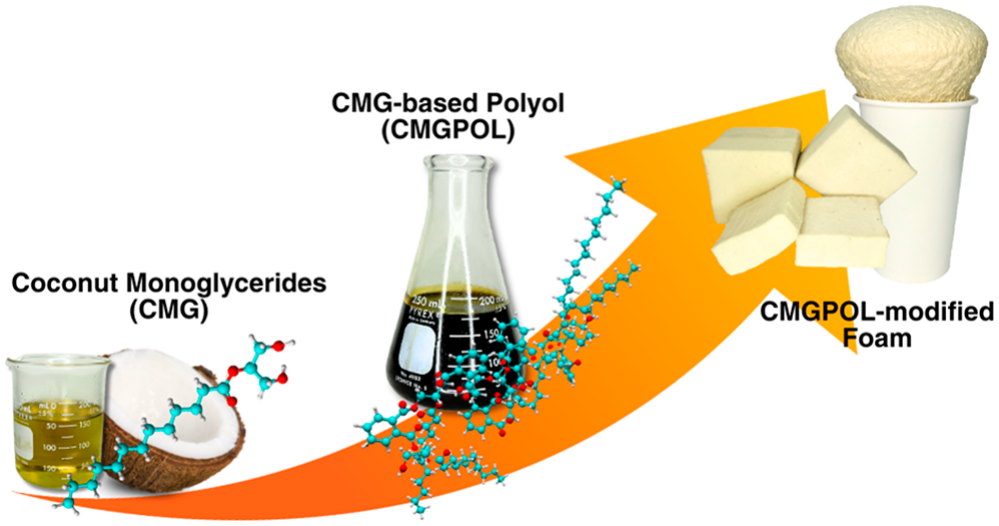

Coconut oil, a low-molecular-weight vegetable oil, is virtually unutilized as a polyol material for flexible polyurethane foam (FPUF) production due to the high-molecular-weight polyol requirement of FPUFs. The saturated chemistry of coconut oil also limits its compatibility with widely used polyol-forming processes, which mostly rely on the unsaturation of vegetable oil for functionalization. Existing studies have only exploited this resource in producing low-molecular-weight polyols for rigid foam synthesis. In this present work, high-molecular-weight polyester polyols were synthesized from coconut monoglycerides (CMG), a coproduct of fatty acid production from coconut oil, via polycondensation at different mass ratios of CMG with 1:5 glycerol:phthalic anhydride. Characterization of the CMG-based polyol (CMGPOL) products showed number-average molecular weights between 1997 and 4275 g/mol, OH numbers between 77 and 142 mg KOH/g, average functionality between 4.8 and 5.8, acid numbers between 4.49 and 23.56 mg KOH/g, and viscosities between 1.27 and 89.57 Pa·s. The polyols were used to synthesize the CMGPOL-modified PU foams (CPFs) at 20 wt % loading. The modification of the foam formulation increased the monodentate and bidentate urea groups, shown using Fourier transform infrared (FTIR) spectroscopy, that promoted microphase separation in the foam matrix, confirmed using atomic force microscopy (AFM) and differential scanning calorimetry (DSC). The implications of the structural change to foam morphology and open cell content were investigated using a scanning electron microscope (SEM) and gas pycnometer. The density of the CPFs decreased, while a significant improvement in their tensile and compressive properties was observed. Also, the CPFs exhibited different resiliency with a correlation to microphase separation. These findings offer a new sustainable polyol raw material that can be used to modify petroleum-based foam and produce flexible foams with varying properties that can be tailored to meet specific requirements.

## Introduction

The polyurethane industry has been regarded as having expansive application and versatility in practical use, such as foams, coatings, elastomers, adhesives, and the like. Polyols, a fundamental component of polyurethane (PU) production, are primarily derived from petroleum products. A polyol contains hydroxyl groups or other isocyanate-reactive compounds needed to form the urethane linkages of a PU material. With the threat of fossil oil depletion, substantial environmental impacts, and economic precarity, there is a growing need for sustainable alternatives to petroleum-derived polyols. Vegetable oils have become a significant potential source of biobased polyols or biopolyols due to their excellent and vast properties, such as unique reactive sites and structures that can be modified and converted into suitable monomers.^1,2^ Numerous vegetable oils including castor oil,^3,4^ soybean oil,^5−7^ palm oil,^8^ corn oil, canola oil, rice bran oil, olive oil, grapeseed oil, linseed oil,^4^ rapeseed oil,^9^ and all their derivatives, have already been employed in polyol synthesis for various applications. These vegetable oils contain varying degrees of unsaturation in their fatty acids, allowing for their modification and customization for individual applications.^10^ Several polyols derived from vegetable oils are already commercially available, primarily for rigid and flexible PU foam applications.^11^ Most of these polyols are from soybean and castor oil under major global producers.

Biobased polyols for FPUFs are not as extensively studied as their rigid counterpart due to the constraints on the extent of biobased polyol replacement in the foam formulation without adverse effects on the mechanical properties of the foam. Thus, modification of the FPUF formulation via partial substitution of biobased polyols has become a widely accepted strategy in producing flexible foams. This technique offers significant advantages not just in lessening the use of petroleum-based materials but also in its opportunity to tailor the resulting properties by different blends of biobased and petroleum-based polyols to achieve specific requirements for an application.^12^ This is owed to the dependency of foam properties on the polyol composition.^13^

For vegetable oil based polyols to be viable replacements for petroleum-based polyols, they must meet essential chemical property requirements such as molecular weight, OH number, and functionality.^14^ Vegetable oils (VOs) are inherently low-molecular-weight compounds and possess some degree of acidity due to the presence of free fatty acids. Also, most vegetable oils do not contain hydroxyl groups, thus necessitating their processing to incorporate this functionality. The most frequently used method in polyol synthesis from vegetable oils is epoxidation, followed by ring opening.^10^ Previous studies have used this process to produce polyols from castor, soybean, palm, and rapeseed oils.^3,5,8,9^ The vital part of vegetable oil involved in this process is the presence of unsaturated fatty acids, whose double bonds are converted to epoxides or oxiranes and subsequently undergo ring opening with alcohol and inorganic acids or by hydrogenation to produce hydroxyl groups. Other processes that target the unsaturation of the oil to produce a polyol include ozonolysis, thiol–ene coupling, reduction/hydroformylation, and metathesis.^10^ Recently, more synthesis routes for polyester polyol production have been developed including polycondensation.^15^ This reaction between diols and diacids typically requires high temperature and pressure, including the use of solvents.^16^ With respect to the use of vegetable oils, hydroxyl (OH) groups are needed, which can be achieved through glycerolysis.

Coconut oil is among the VOs produced on multiple continents across the globe. Primary producers are found in Asia and South America, while some minor sources can be found in portions of Africa and Oceania. The coconut oil market was valued at USD5.3B in 2021 with a compound annual growth rate of 5.7%,^17^ maintaining a stable global production of 3.70 million MT from 2021 to the present.^18^ This suggests that this VO is a relevant material with the capacity to sustain other applications. However, coconut oil and its derivatives have not been commonly used in polyol synthesis due to their saturated nature, making them incompatible with the previously mentioned processes.^10^ Coconut oil is known to contain 80–90% saturated fat with lauric acid as the predominant fatty acid at 47%,^19^ along with short and medium chain acids (C_8_–C_14_) of around 30%, while the rest are longer chains (C_16_–C_18_).^20^ In order to be a viable prospect for polyol use, a material must possess functional groups that will serve as reaction sites.

A derivative of coconut oil that satisfies said functional requirements is coconut monoglycerides (CMG). CMG is a coproduct of fatty acid production^21^ and can also be derived from coconut oil via glycerolysis. It contains two OH functionalities that meet the requirements of polycondensation as a process to produce polyols.

Few existing studies that used CMG include its reaction with polycarbonate in a microwave-assisted process to produce low-molecular-weight polyols^22^ and its use in the recycling of waste PU and polycarbonate to produce polyols for rigid foam.^23^ In other studies, CMG was utilized in the production of a solid ecoresin for superhydrophobic coating^24^ and a PU coating with modified nano TiO_2_ with improved thermal stability and gloss characteristics.^25^ Another study focused on producing new alkyd resin surface coating binders from CMG.^26^ The low-molecular-weight CMG-based polyols from the reported works ranged between 213 and 1322 g/mol.^22−26^ These studies were only for rigid PU applications and surface coatings, with the former requiring polyols with molecular weights of 150–1000 Da,^27^ making CMG with an estimated average molecular weight of 274.4 Da (based on monolaurin molecular weight; assumed high purity CMG) suitable for use without further polymerization.

However, the extremely low-molecular-weight of CMG does not satisfy the requirements for FPUF applications. For instance, commercially available petroleum-based and soybean oil based polyols for this application produced by Dow, VORANOL,^28^ Renuva,^29^ and Cargill^30^ have molecular weights roughly ranging from 700 to 5000 g/mol, OH numbers of 30–250 mg KOH/g, and functionality of 2–3. In general, polyols for FPUF applications must have a molecular weight between 1000 and 6000 g/mol, an OH number between 28 and 160 mg KOH/g, and a functionality of 2–3.^27,31,32^

For CMG or other vegetable oil based materials to compete with the mentioned products and other commercially available polyols, it must meet the mentioned structural specifications of the fossil competitors.^14,25,27−30^ In this regard, there is still no reported nor published research involving the utilization of CMG in polymerization to produce polyols with properties tailored to satisfy the requirements of FPUFs. Hence, the researchers of this study were compelled to investigate the potential of CMG as a polyol raw material specifically for FPUF applications. This research presents a novel polyester polyol synthesized from CMG with high-molecular-weight and properties suitable for FPUFs. Uncatalyzed and solvent-free polycondensation was employed. The physicochemical, structural, and thermal properties of the resulting polyols were analyzed by different ASTM methods, FTIR techniques, and thermal analyses. The viability of the polyols for flexible foam applications was also demonstrated by synthesizing foam modified with the CMG-based polyols. Foam characterizations included physical and mechanical testing, FTIR investigation, morphological inspection, and thermal analyses.

## Methodology

### Materials

CMG and glycerol were obtained from Chemrez Technologies, Inc. (Quezon City, Philippines), with properties listed in Table 1. AR-grade phthalic anhydride (PA) and stannous octoate were from Sigma-Aldrich (St. Louis, MO, USA), polymeric MDI (pMDI), VORANOL 4701 and VORASURF SZ 1952 were from Dow Chemical Company (Hayward, CA, USA), POLYCAT 8 and DABCO BL-17 were from Evonik Industries (Germany), and NIAX A-1 and L-580 were from Momentive Performance Materials Inc. (Niskayuna, NY, USA) as provided by Chemrez Technologies, Inc. All materials were used without further purification steps.

**Table 1:** Properties of Raw Materials Coconut Monoglycerides (CMG) and Glycerol

properties	CMG	glycerol
OH number, mg KOH/g	313	1818
free fatty acid, %	0.001	1.0 max
purity, %	98	99.5
appearance	off-white, semisolid paste	colorless, viscous liquid
source material	coconut oil	coconut oil

### Polyol Synthesis

Different polyol formulations consisting of glycerol, PA, and CMG were weighed and mixed in an Erlenmeyer flask according to the mass ratios listed in Table 2. The starting ratio of reactants was determined stoichiometrically, and subsequent ratios were based on increasing excess of CMG by a molar increment. The flask was placed on a hot plate with a magnetic stirrer, and a thermometer clamped on an iron stand was inserted in the flask. The reaction was done at 120 °C for 30 min with constant stirring at 1000 rpm. Then the temperature was increased to 180 °C, and the reaction was allowed to proceed for 3 h with constant stirring at 1000 rpm. After the reaction, the products were subjected to vacuum drying for 2 h at 160 °C. The final polyol products, referred to as CMG-based polyol (CMGPOL), followed by the mass ratio of CMG in the polyol formulation, were dark brown liquids that were physically stable at room temperatures. They were stored in tightly sealed containers.

**Table 2:** Mass Ratios for Coconut Monoglycerides-Based Polyols (CMGPOL) Products

CMGPOL series	CMG:glycerol:PA mass ratios
CMGPOL-8	8:1:5
CMGPOL-12	12:1:5
CMGPOL-16	16:1:5
CMGPOL-20	20:1:5
CMGPOL-24	24:1:5

### Foam Synthesis

Foams were synthesized using the CMGPOL products at a constant loading of 20 wt % to determine their effect on the properties of resulting foams. Also, a petroleum-based control foam was synthesized as a reference material. Table 3 lists the components of the foam systems. In the foaming process, the B-side components were weighed in a cup mold and stirred vigorously at 2000 rpm for 1 min. Then, the A-side component was added to the mixture and stirred at the same speed for 15 s. The reaction mixture was allowed to expand in the open cup mold and cured at ambient conditions for 24 h.

**Table 3:** Foam Formulation for CMGPOL-Modified Polyurethane Foams (CPFs) and Petroleum-Based Control Foam

	flexible foam formulation (PPHP)^a^	
components	control	CPFs
A-side		
pMDI (isocyanate index)	65	
B-side		
CMGPOLs	0	20
VORANOL 4701	100	80
water	3.5	
POLYCAT 8	0.5	
stannous octoate	0.25	
NIAX A-1	0.25	
DABCO BL-17	0.5	
VORASURF SZ 1952	1	
NIAX L-580	0.5	

a Parts per hundred parts of polyol.

### Analytical Methods

#### Gel Permeation Chromatography (GPC)

The number average, weight average molecular weight (*M*_n_ and *M*_w_), and molecular weight distribution (*M*_w_/*M*_n_) of the polyols were determined using Shimadzu Prominence gel permeation chromatography (GPC) system equipped with a refractive index detector (RID-20A) and a Shodex GPC column (KF-803L) (Shimadzu Corp., Kyoto, Japan). The data were collected by LabSolutions software. Tetrahydrofuran (THF) was used as the mobile phase at a flow rate of 1.0 mL/min for 15 min. The operating parameters employed were an injection volume of 50 μL and a column and detector temperatures of 40 °C. Seven polystyrene (PS) standards from Shimadzu Philippines Corp. were used for molecular weight distribution calibration (700–18000 Da).

#### Attenuated Total Reflectance–Fourier Transform Infrared (ATR–FTIR)

Attenuated Total Reflectance–Fourier Transform Infrared (ATR–FTIR) spectroscopy analysis was conducted for the polyol and foam samples using a Shimadzu IRTracer-100 spectrometer equipped with QATR-10 single reflection ATR accessory with a diamond crystal (Shimadzu Corp., Kyoto, Japan). The wavenumber range included was 500–4000 cm^–1^ at a resolution of 4 cm^–1^.

#### Chemical and Physical Tests

The OH number, acid number, and viscosity of the polyols were determined according to ASTM D4274,^33^ ASTM D1980,^34^ and ASTM D4878,^35^ respectively. A Brookfield DV3T Rheometer (AMETEK Brookfield, Middleborough, MA) was used to determine the viscosity.

#### Thermal Analysis

Differential scanning calorimetry (DSC) test was performed using PerkinElmer DSC 4000 with a (1020 TA) workstation (Waltham, MA). The thermal investigation of the polyols was done with a temperature range of −75 to 200 °C at a heating rate of 10 °C/min in a 20 mL/min nitrogen atmosphere, while that of the foams was done with a temperature range of −50 to 250 °C at a heating rate of 10 °C/min in a 20 mL/min nitrogen atmosphere.

#### Thermogravimetric Analysis

The thermal stability of the polyols and foams was investigated using Shimadzu DTG-60H (Shimadzu Corp., Kyoto, Japan). The thermograms of the polyols were recorded at a temperature range of 50–500 °C. The heating rate was set to 10 °C/min with a nitrogen flow rate of 40 mL/min. The thermograms of the foams were recorded at a temperature range of 50–700 °C with a heating rate of 10 °C/min and a nitrogen flow rate of 40 mL/min.

#### Foam Characterizations

Atomic force microscopy (AFM) Shimadzu SPM-9700HT (Shimadzu Corp., Kyoto, Japan) was used to evaluate the hard–soft domains phase separation of the foam. The instrument was operated at a scan rate of 1.0 Hz in a noncontact mode with Nanoworld NCHR-10 Pointprobe-Silicon SPM-Sensor phase cantilever with the following specifications: 4 μm thickness, 125 μm length, 30 μm width, 320 kHz resonance frequency, and 42 N/m force constant. Scanning electron microscopy (SEM) images of the foam samples were obtained using HITACHI SU-1510 (Japan) at 35× magnification. The samples were coated with a gold–palladium alloy for 1 min at 10 mA and a sample-target distance of 15 mm. The cell size distribution of the foams was analyzed using ImageJ. The open cell content of the foam samples was determined according to ASTM D6226^36^ using Quantachrome Ultrapyc 1200e automatic gas pycnometer (Germany). The mechanical and physical properties of the foam were tested using Universal Testing Machine AGS-X Series (Shimadzu Corp., Kyoto, Japan) with ASTM standard tests. Density, compression force deflection (CFD) at 50% deformation, tensile strength, and ball rebound resilience tests were conducted according to ASTM D3574^37^ Tests A, D, E, and H, respectively.

## Results and Discussion

### Polycondensation Prcess

**Figure 1:**
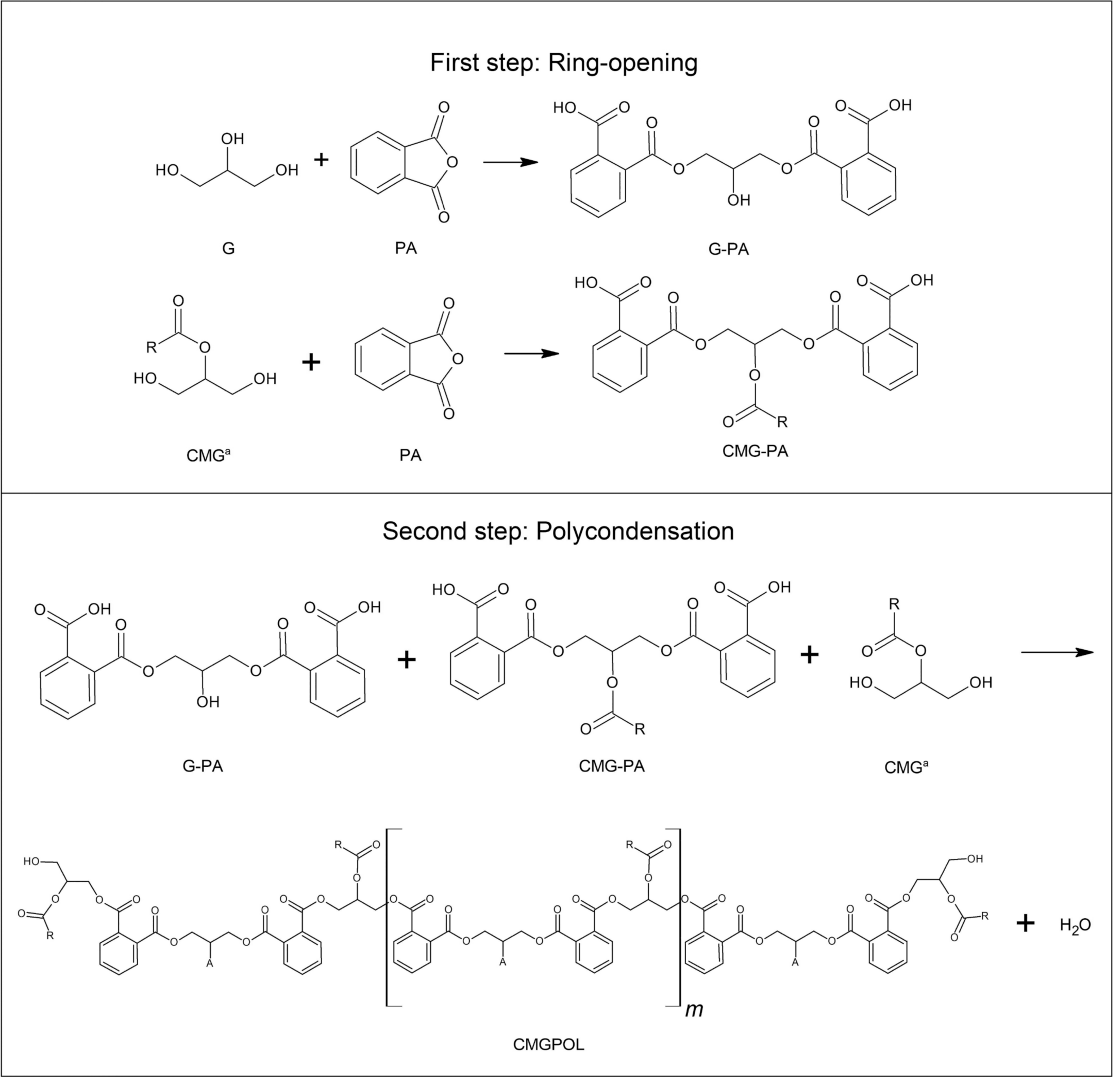
Reaction mechanism between coconut monoglycerides (CMG), glycerol (G), and phthalic anhydride (PA) without cross-linking to produce CMG-based polyol (CMGPOL). Ring-opening and polycondensation were conducted at 120 and 180 °C, respectively. R represents the fatty acid carbon chain, while A stands for either a hydroxyl group from glycerol or a fatty acid group of coconut oil. ^a^β-CMG.

This work employed polycondensation to yield a high-molecular-weight polyol from CMG to meet the FPUF application requirements. As previously reported by published studies on vegetable oil based polyols via polycondensation,^2,38^ the reaction has two distinct steps (Figure 1). First is the initial reaction between PA and the primary hydroxyl groups in both CMG and glycerol at slightly lower temperatures, and second is the polycondensation reaction between the carboxyl groups in PA and the remaining hydroxyl groups.

The first step readily occurs around 120–130 °C,^2,38^ forming two intermediates: glycerol-PA (G-PA) and CMG-PA. The R group in CMG represents a carbon chain of fatty acids in coconut oil, primarily C12 for lauric acid. The reaction between PA and hydroxyl groups opens the anhydride ring on PA, creates an ester bond, and leaves one free carboxyl group on PA. This mechanism is supported by the absence of water generation during the reaction. At this condition, primary hydroxyl groups were expected to participate in the reaction due to their higher reactivity at temperatures around 100–150 °C than the secondary hydroxyl groups.^39^ Thus, the experiment employed 120 °C for the dissolution and ring opening of PA. The second step involves the polycondensation process, producing polyester polyol with water as a byproduct. The temperature required for thermal polymerization to occur is within the range of 130–200 °C.^40^ According to two related studies,^2,38^ a successful polycondensation was observed between 180 and 200 °C. Moreover, due to the smoke point of coconut oil at 191 °C,^41^ a temperature of 180 °C was maintained for this step.

As shown in Figure 1, both CMG-PA and G-PA would compete to react with CMG, resulting in the formation of a polyester polyol, CMGPOL, and water as a byproduct. Different mass fractions of CMG would constitute a difference in the structure of the polyol, as monomeric composition, or the nature of the monomer components, significantly impacts the molecular structure of the resulting product.^42^ The A groups in CMGPOL denote either the fatty acid group in CMG or the secondary hydroxyl group of glycerol. CMG-PA component would be the main constituent of chain lengthening due to the higher concentration of CMG in the mixture than glycerol. Aside from linear step growth, the G-PA component could also participate in cross-linking through its secondary hydroxyl group. Its likelihood is inversely correlated to the amount of CMG in the mixture.

However, Figure 1 illustrates the suggested reaction mechanism, assuming that excess CMG was added to the mixture to avoid cross-linking. Further, CMG exists in two distinct forms depending on the transesterified hydroxyl group on glycerol: α-CMG and β-CMG for the primary and secondary hydroxyl groups, respectively. The sample used in this study has 60.53% β-CMG content. Thus, the proposed mechanism in Figure 1 reflects the β form of CMG as this is most abundant in the mixture.

### Gel Permeation Chromatography (GPC) of Polyols

**Figure 2:**
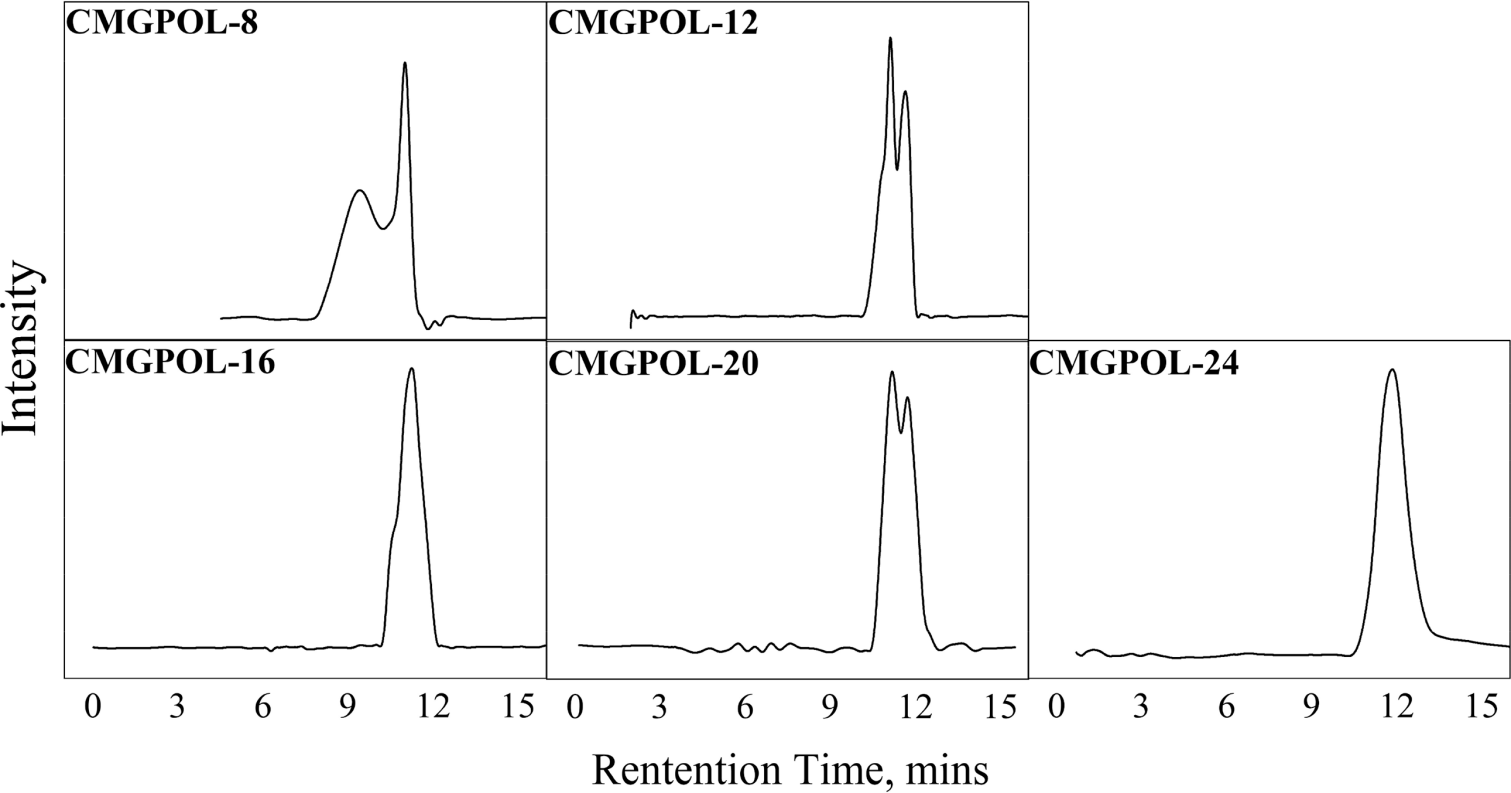
GPC chromatograms of coconut monoglycerides-based polyols (CMGPOL) derived from the reaction of CMG, glycerol, and phthalic anhydride at different CMG mass ratios.

Table 4 summarizes the GPC results of the polyols estimated through a calibration curve from seven polystyrene standards. These values hint that the molecular weights of all CMGPOLs satisfy the target 1000–6000 g/mol for flexible foam application.^27,31^Figure 2 depicts the corresponding CMGPOL chromatograms, showing that a higher CMG mass ratio led to longer retention times. This suggests that the molecular weight of the polyols decreases with increasing CMG loading. This correlation is evident in the GPC numerical results shown in Table 4. CMGPOL-8 exhibited a characteristic broad peak between a retention time range of 8–10 min, signaling the presence of relatively higher molecular weights than its other distinct peak at about 11 min, indicated in Figure 2. The broad peak suggests the occurrence of cross-linking in the reaction, resulting in an exceptionally high-molecular-weight polyol owing to the lower CMG content in the mixture. As illustrated in Figure 1, the excess CMG provided the OH groups needed by the two intermediate products, CMG-PA and G-PA, to react with and polymerize during the second step of the reaction. The presence of less CMG in the CMGPOL-8 formulation would increase the likelihood of cross-linking as the secondary OH group of glycerol would significantly contribute to the available reactive sites leading to cross-linking. The same phenomenon has been explained by Chen et al.^39^ wherein fewer primary OH groups in the reaction mixture controlled the degree of polymerization versus cross-linking in favor of the latter. Thus, as the CMG loading increases, the speculated cross-linking density decreases, reflected as a decrease in molecular weights.

**Table 4:** GPC Results of Different Coconut Monoglycerides-Based Polyol Products (CMGPOL)

CMGPOL	*M*_n_ (Da)	*M*_w_ (Da)	M_*z*_ (Da)	PDI^a^
CMGPOL-8	4275	5361	7253	1.25
CMGPOL-12	3197	3799	4586	1.19
CMGPOL-16	2224	2519	2732	1.13
CMGPOL-20	2158	2462	2681	1.14
CMGPOL-24	1997	2322	2544	1.16

a PDI, polydispersity index. PDI = *M*_w_/*M*_n_

The polydispersity index (PDI) of the samples was roughly between 1.13 and 1.25. The PDI is a value that describes the molecular weight distribution of a compound with an ideal value of 1.0, meaning that the polyols are monodispersed or that the polyol molecules have the same molecular weight.^43^ According to Ionescu, polyols obtained from polycondensation generally have a PDI of 2.5–2.8.^32^ Despite this general rule, several studies have successfully produced polyols with PDIs outside this range, including aliphatic copolyesters with PDI between 1.06 and 3.30 via combined ring opening and polycondensation,^44^ unsaturated hydroxypolyesters with PDI between 1.3 and 17.5 via direct polycondensation and transesterification,^45^ and poly(polyol sebacate) with PDI between 1.6 and 4.4 via polycondensation.^46^ In parallel with the decreasing trend of molecular weight relative to increasing CMG mass ratio, a decreasing trend in PDI can also be noted, albeit it increased back starting with CMGPOL-20. This increase may be attributed to the excess CMG, producing oligomers with diverse sizes.

### IR Spectra of Polyols

**Figure 3:**
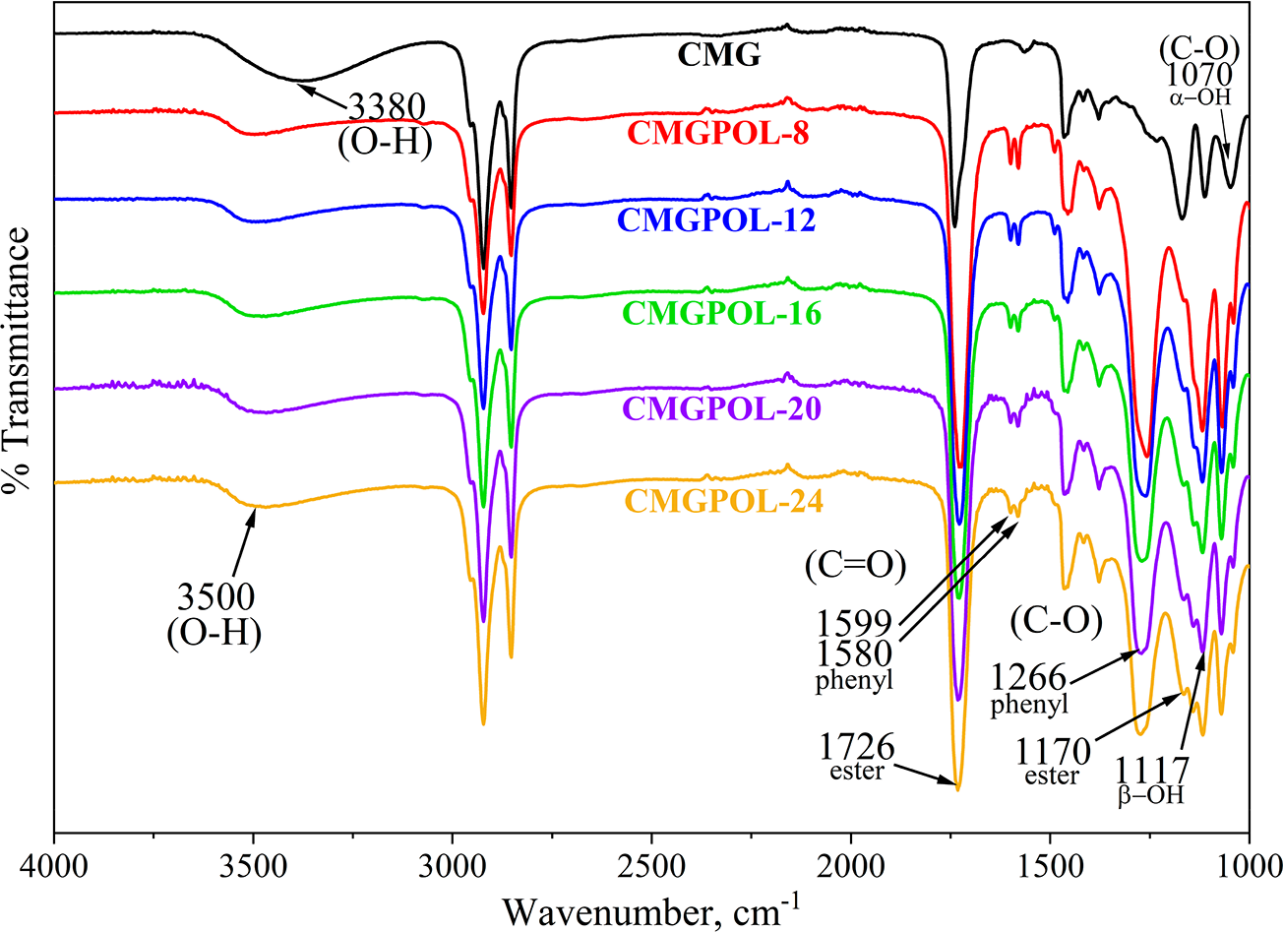
FTIR spectra of coconut monoglycerides (CMG) and CMG-based polyols (CMGPOL) at different CMG mass ratios.

Figure 3 compares the IR spectra of the synthesized polyols and CMG. All synthesized polyols displayed identical characteristic peaks but with apparent differences when compared with CMG. The O–H stretching vibrations of the CMGPOLs exhibited moderate peaks at 3500 cm^–1^. In contrast, CMG displayed a broad O–H stretching peak at 3380 cm^–1^ owing to its high OH value. The drop in the O–H peak intensity of the CMGPOLs with respect to CMG suggested their consumption during the reaction. It can also be noted that relative to each CMGPOL, the O–H peak has an increasing trend. This observation implies the notion of cross-linking at a lower CMG mass ratio. A distinct shift of the hydroxyl peaks toward higher wavenumbers was apparent in the CMGPOLs compared to CMG at the same bandwidth. This shift can be explained by the diminishing strength of hydrogen bonding interactions between O–H groups, likely due to the decrease in its number and stearic hindrances. The opposite is true with increasing number of hydroxyl groups that tend to shift the peak toward the lower wavenumber, also called the blue shift.^47−49^ Moreover, the sharpening of the ester bond C=O stretch bands at 1726 cm^–1^ in the CMGPOLs relative to CMG provided another indication of the formation of ester bonds on the product chain. These peaks decrease as the CMG mass ratio increases. The absence of peaks for anhydride C=O stretch at 1818 cm^–1^, strong and very broad carboxylic acid O–H stretch at 3300–2500 cm^–1^, and carboxylic acid dimer C=O stretch at 1720–1706 cm^–1^ evinced the near completion of esterification reaction, thus producing hydroxyl-terminated products. The narrow and sharp peaks within the carboxylic acid O–H stretch range correspond mainly to the stretching vibrations of C–H groups. Twin peaks at 1599 and 1580 cm^–1^ are distinctive on the spectra of the CMGPOLs. These bands represent the C=C stretch of cyclic alkenes in PA and the conjugation of the C=O stretch with the phenyl ring of PA.^2^ In contrast, these peaks were absent from the spectra of CMG. Another peak characteristic in CMGPOLs that is missing in CMG is the pronounced C–O stretch of aromatic esters at 1266 cm^–1^. The C–O stretching of the ester bonds in the fatty acid of CMG is also present in all synthesized CMGPOLs at 1170 cm^–1^, albeit to a much lesser degree. Moreover, the C–O stretch of primary and secondary alcohols in CMGPOLs is reflected at 1070 and 1117 cm^–1^, respectively. This result indicates that a significant amount of primary hydroxyl groups is present in the polyol products, a preferred type of functionality due to their higher reactivity. These bands are equivalent to the peaks at 1046 and 1117 cm^–1^ in the CMG spectrum.

### Physicochemical Characteristics of Polyols

**Figure 4:**
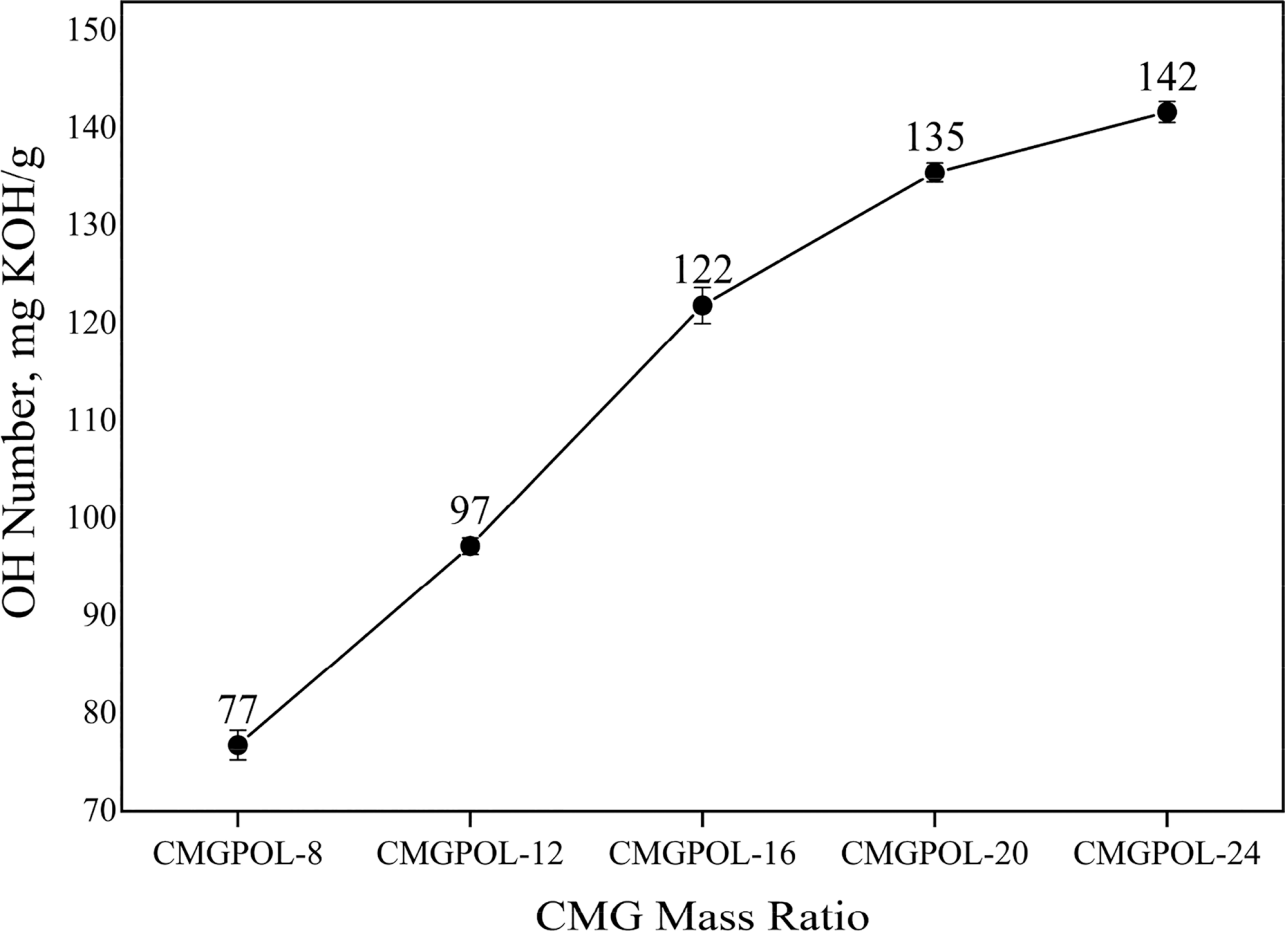
Effect of changing coconut monoglycerides (CMG) mass ratio on the OH number of CMG-based polyols (CMGPOL).

The physicochemical properties investigated in this section are the OH number, functionality, acid number, and viscosity of the CMGPOLs. The presence of OH groups as reactive sites for isocyanates during the foaming process is denoted as the OH number.^32^ The average OH number of all synthesized polyols ranged from 77 to 142 mg KOH/g, which is within the standard requirement for flexible foam polyols.^27^ This apparent decrease in OH number of the CMGPOLs compared with the OH numbers of CMG and glycerol proved the occurrence of the esterification reaction between both CMG and glycerol with PA. A direct relationship between the OH number of the polyols and CMG mass ratio is observed, as shown in Figure 4. This trend coincides with the GPC results and FTIR findings, wherein lower CMG loading resulted in lower OH number and higher molecular weights. This agrees with the postulated cross-linking of the polyol at lower CMG mass ratios.

The functionality is among the most crucial characteristics of polyols.^32^ It refers to the number of OH groups per molecule. The number-average functionality of CMGPOLs was calculated from their OH number and *M*_*n*_ as follows:^14,32^
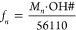
1where *M*_*n*_ is the number-average molecular weight, and OH# is the OH number of the sample. Results in Table 5 show that the *f*_*n*_ of the polyol products is between 4.8 and 5.8. These values are higher than the general functionality of polyols for flexible foam applications. Despite this, several studies have successfully utilized polyester polyols with higher functionality (*f* > 3) to produce viscoelastic foam, a type of flexible foam known for its slow recovery/resilience characteristics.^50^ Rojek and Prociak used vegetable oil based polyol with a functionality of 5.3 mixed with a petroleum-based polyol.^9^ The outcome was a flexible foam with lower resiliency. The lower resiliency is due to high polyol functionality, indicating a high number of oligomers and variations in chain mobility. In effect, the foam had better energy absorption and was thus better suited for viscoelastic foam applications. Moreover, polyols with higher functionality give higher cross-linking density and urea formation during the foaming process, improving the tensile strength of the foam. Similarly, higher functionality decreases shape recovery, thus increasing its viscoelastic quality.^51^

**Table 5:** Functionalities of Coconut Monoglycerides-Based Polyols (CMGPOL)

CMGPOL	functionality
CMGPOL-8	5.8
CMGPOL-12	5.5
CMGPOL-16	4.8
CMGPOL-20	5.2
CMGPOL-24	5.0

**Figure 5:**
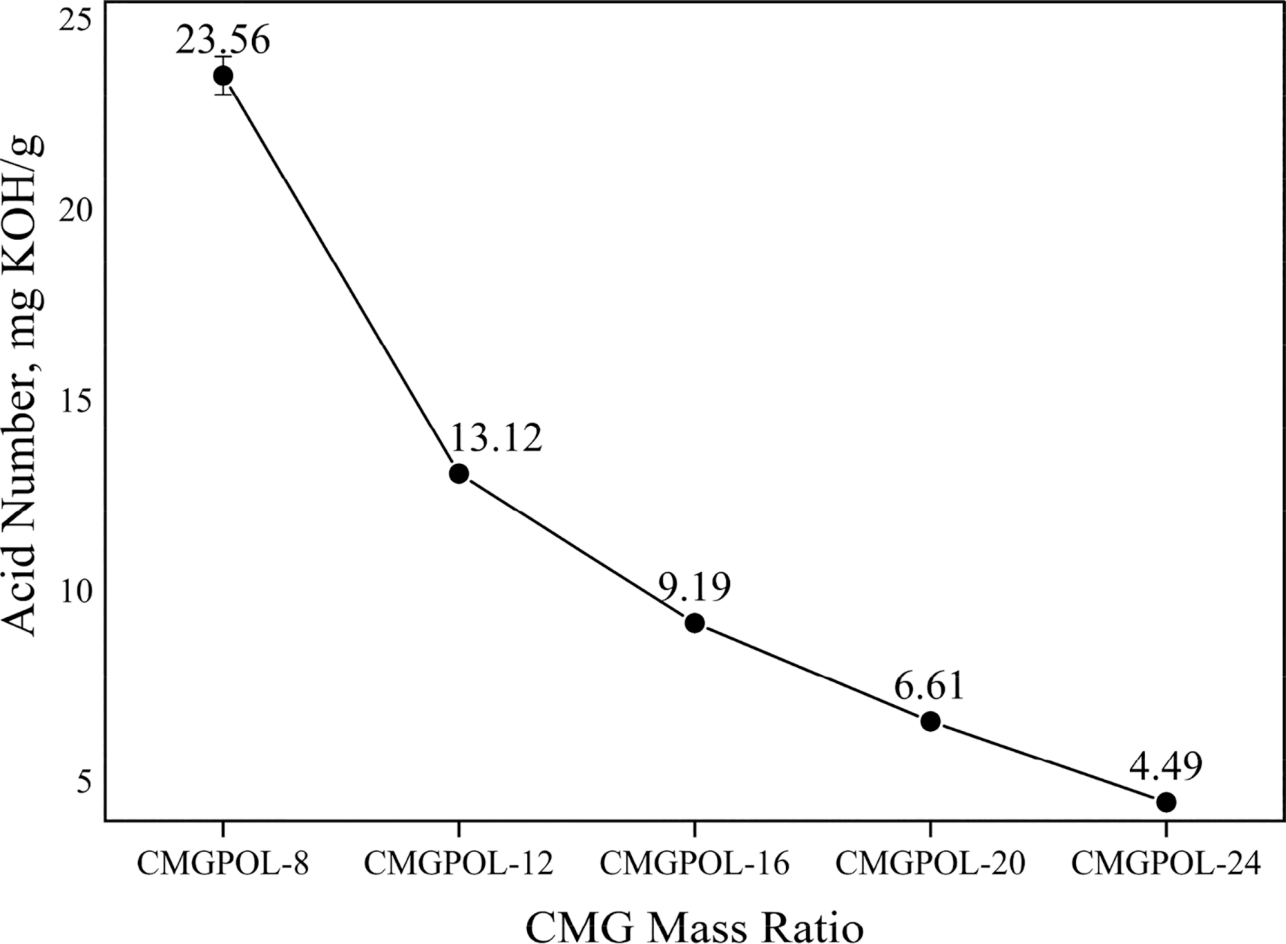
Effect of changing coconut monoglycerides (CMG) mass ratio on the acid number of CMG-based polyols (CMGPOL).

Another essential characteristic of polyols, the acid number, negatively impacts their reactivity during PU synthesis.^32^ Thus, this value needs to be minimized. The average acid values of the CMGPOLs were recorded to be between 4 and 24 mg KOH/g, shown in Figure 5. PA is the main component that contributed to the acid content of the samples due to its acidic nature, with an acid number of 757.5 mg KOH/g.^52^ Based on the results, the decrease in the acid number may be attributed to the consumption of PA in the esterification reactions as the reaction progressed. Three polyols showed an acid number of less than 10 mg KOH/g among the five different formulations.

**Figure 6:**
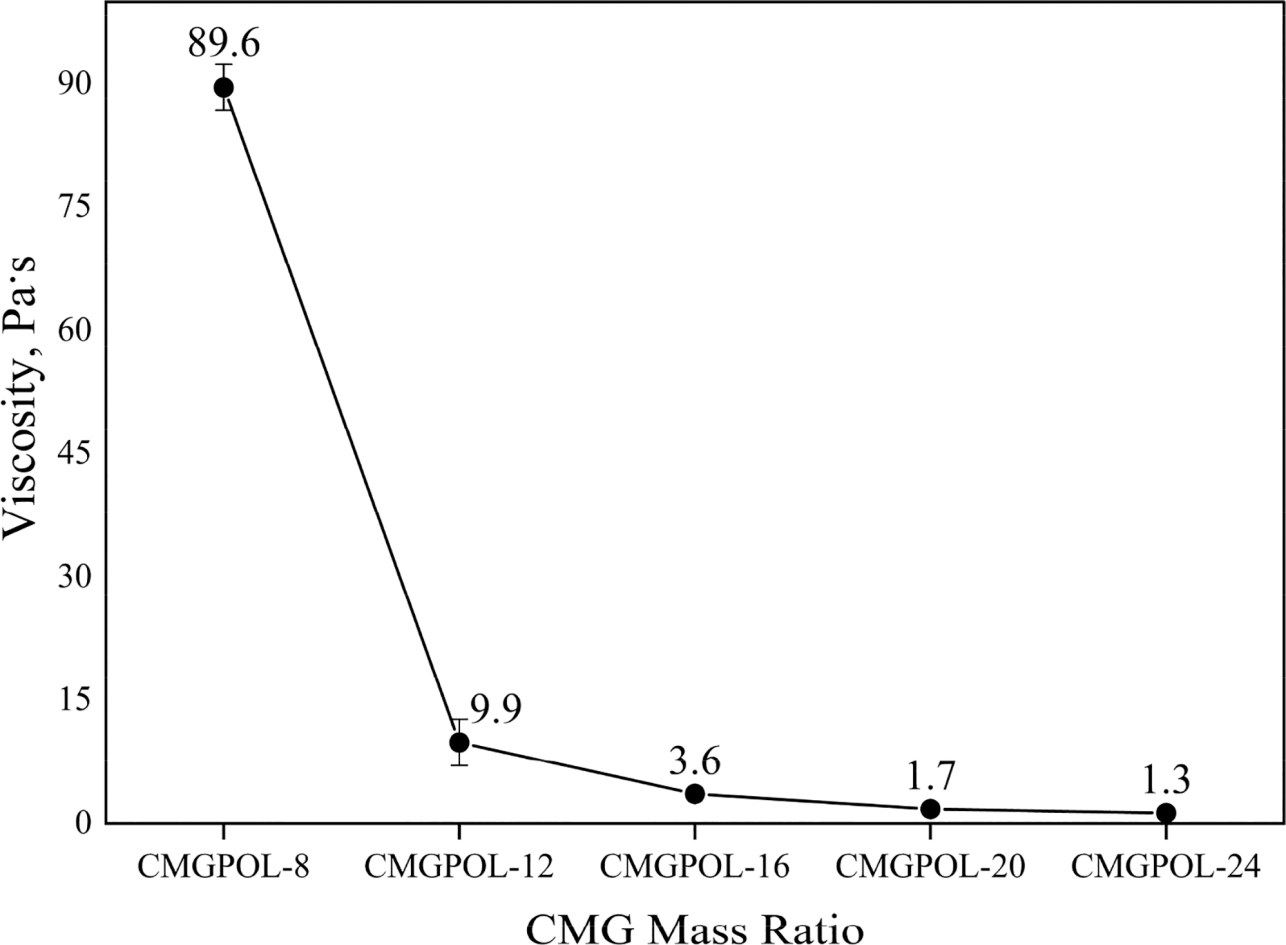
Effect of changing coconut monoglycerides (CMG) mass ratio on the viscosity of CMG-based polyols (CMGPOL).

The dynamic viscosity of the polyols increased with decreasing mass ratio of CMG, as depicted in Figure 6. In other words, an increasing trend in viscosity was noted with increasing relative contents of glycerol. An increasing relative content of glycerol implied an increasing relative content of OH groups in the mixture since glycerol had a much higher OH number than CMG. Thus, the increasing trend in viscosity can be correlated to the increasing OH group content of the mixture, which is directly associated with cross-linking. This observation is consistent with the findings of Aydin et al., in which the viscosities of the products increased with increasing OH group per mass of the sample.^52^ The viscosities of the polyols were also within the preferable viscosity of less than 50 Pa·s,^15^ except for CMGPOL-8.

### Thermal Characteristics of Polyols

**Figure 7:**
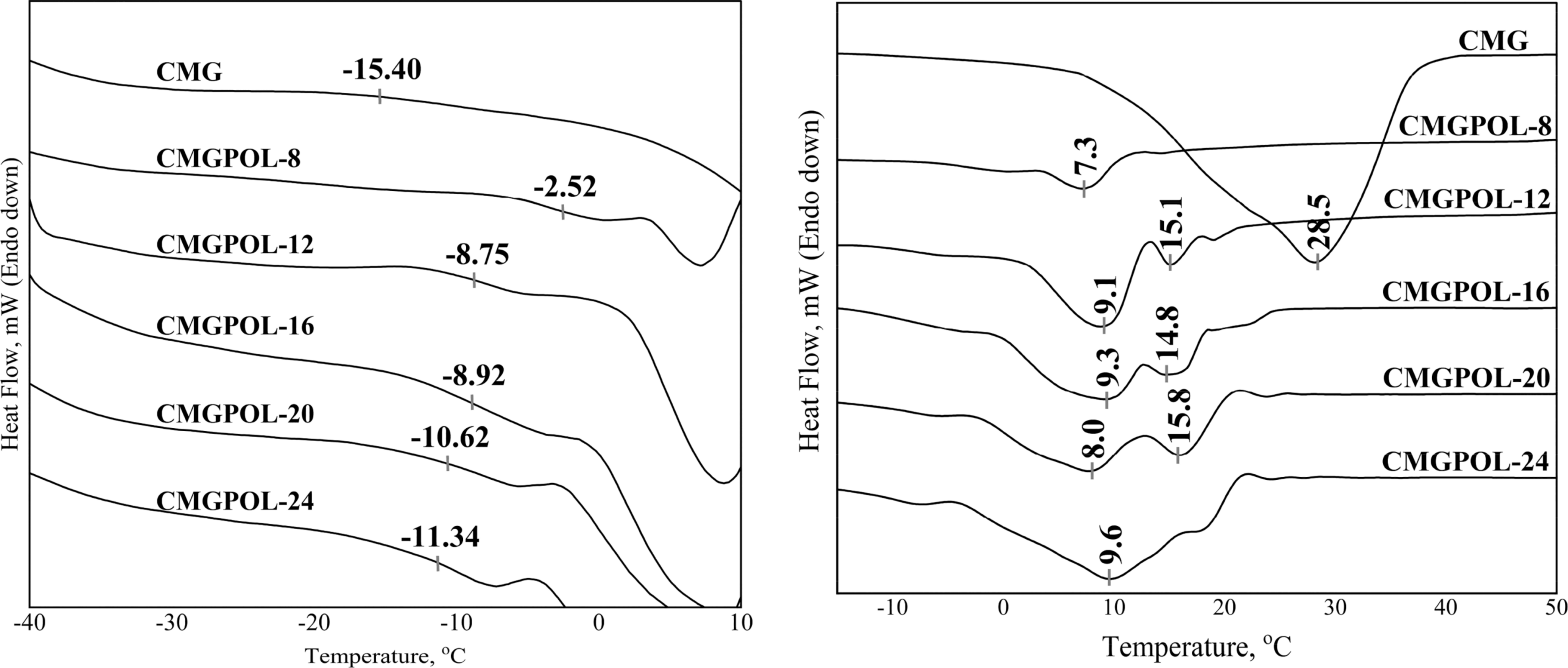
DSC thermogram of coconut monoglycerides (CMG) and synthesized CMG-based polyols (CMGPOL) showing the glass transition temperatures (left) and melting points (right).

Figure 7 depicted the DSC results of the synthesized polyols. Glass transition temperatures, *T*_g_, between −12 °C to −2 °C were recorded in contrast with *T*_g_ of CMG at −15.40 °C. These results suggested that the polyols contained soft segments within their structure. As the amount of CMG added increased, the glass transition temperature of the polyols shifted closer to the glass transition temperature of CMG. This trend can be correlated to the cross-linking density having a direct relationship with glass transition temperature.^14^ Following this trend and correlation, it can be noticed that the *T*_g_ of CMGPOL-8 is significantly higher than the rest of the samples. This indicates a relatively higher degree of cross-linking in CMGPOL-8 than in the other polyols. The addition of CMG decreased the overall ratio of glycerol, a cross-linking agent, thus reducing the relative cross-linking density. Also, considerably lower melting points were recorded for all polyols with melting peaks ca. between 7 and 16 °C. The melting point can be correlated to the degree of polymerization of the polyols. A high melting point (>80 °C) suggests a very low degree of polymerization, and the polyol possesses weak mechanical strength due to high monomer content.^39^ This roughly coincides with the GPC trend that cross-linking increases with decreasing CMG loading. The most relatively cross-linked sample, CMGPOL-8, has the lowest melting temperature due to a relatively high degree of polymerization. CMGPOL-12, -16, and -20 exhibited two distinct melting points that agreed with the GPC chromatogram results in Figure 2. Overall, the results implied a high degree of polymerization and the potential to produce foam with good mechanical strength.

**Figure 8:**
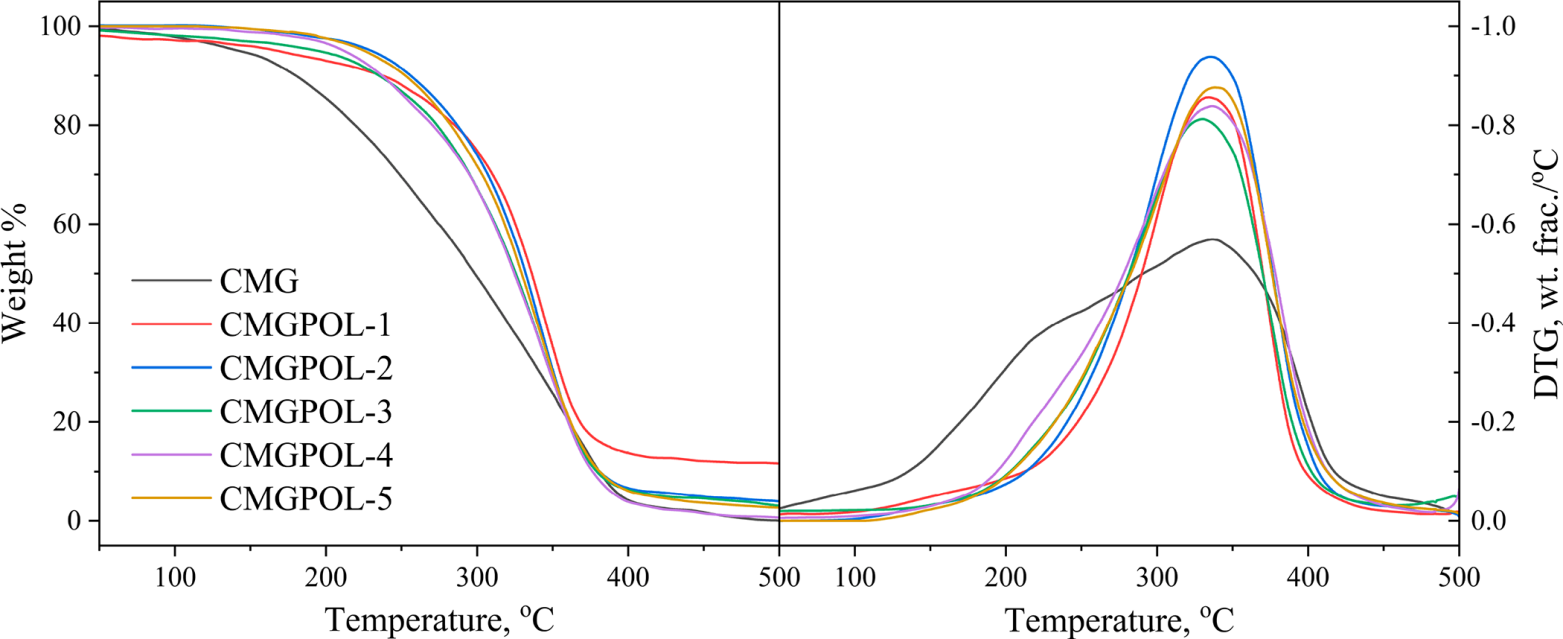
TG and DTG curves of coconut monoglyceride (CMG) and the CMG-based polyols (CMGPOL) at different CMG mass ratios.

The thermogravimetric analyses of synthesized polyol products were performed to investigate the weight loss of the CMGPOLs as a function of temperature. Figure 8 shows the TG and DTG curves of CMG and CMGPOLs. All CMGPOLs exhibited almost similar thermal degradation profiles with one distinct degradation peak at approximately 335 °C, mainly corresponding to the decomposition of the polyol chain. In contrast, the raw material CMG recorded a degradation peak at 230 °C. Relative to CMG, the polyols have increased thermal resistance. This increase in the degradation temperature of the polyols can be attributed to the formation of the polymeric network.^53^

### Foam Characterizations

The CMG-based polyols (CMGPOLs) were utilized to modify flexible PU foam formulations at 20% biobased polyol replacement. This investigation was employed to determine the corresponding effects of the different CMGPOLs on the properties of the resulting foams. This biobased polyol loading of 20 wt % was employed following similar studies that demonstrated improvements in foam properties due to modification with biobased polyols.^9,54^

**Figure 9:**
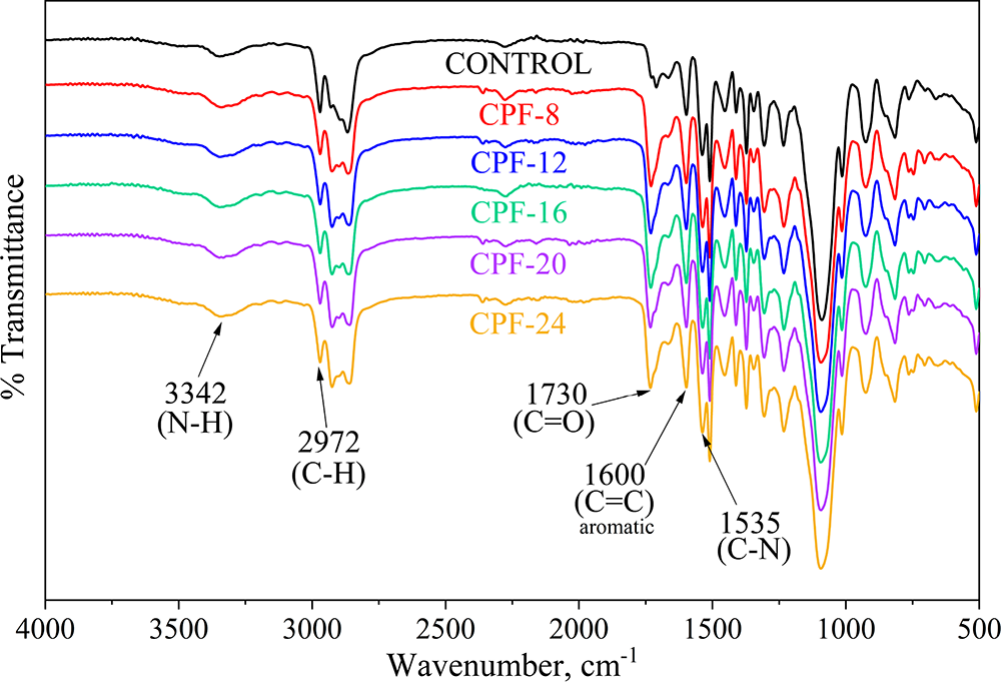
FTIR spectra of CMGPOL-modified polyurethane foams (CPFs) and petroleum-based control foam.

Figure 9 shows the IR spectra of the CMGPOL-modified PU foams (CPFs) and petroleum-based control foam. The key absorption bands are at 3342 cm^–1^ attributed to the N–H bonds stretching vibration of urethane, 2972 cm^–1^ to the C–H bonds of CH_2_, 1730 cm^–1^ to the C=O bonds of urethane linkages and of esters on the polyol chain, 1600 cm^–1^ to the C=C of aromatic rings, and 1535 cm^–1^ to the C–N bonds. The bands mentioned have significantly greater intensity in the CPFs than in the control. This is attributed to the higher OH number of the CMGPOLs, which leads to increased isocyanate requirement. Further, conspicuous augmentation of the C=O and C=C bands can be seen in Figure 9. The increase in these transmission bands of the CPFs is ascribed to the structure of CMGPOL components, specifically the fatty acid ester linkages and benzene ring in phthalic anhydride, respectively. This shows the successful incorporation of the CMGPOLs in the PU matrix.

**Figure 10:**
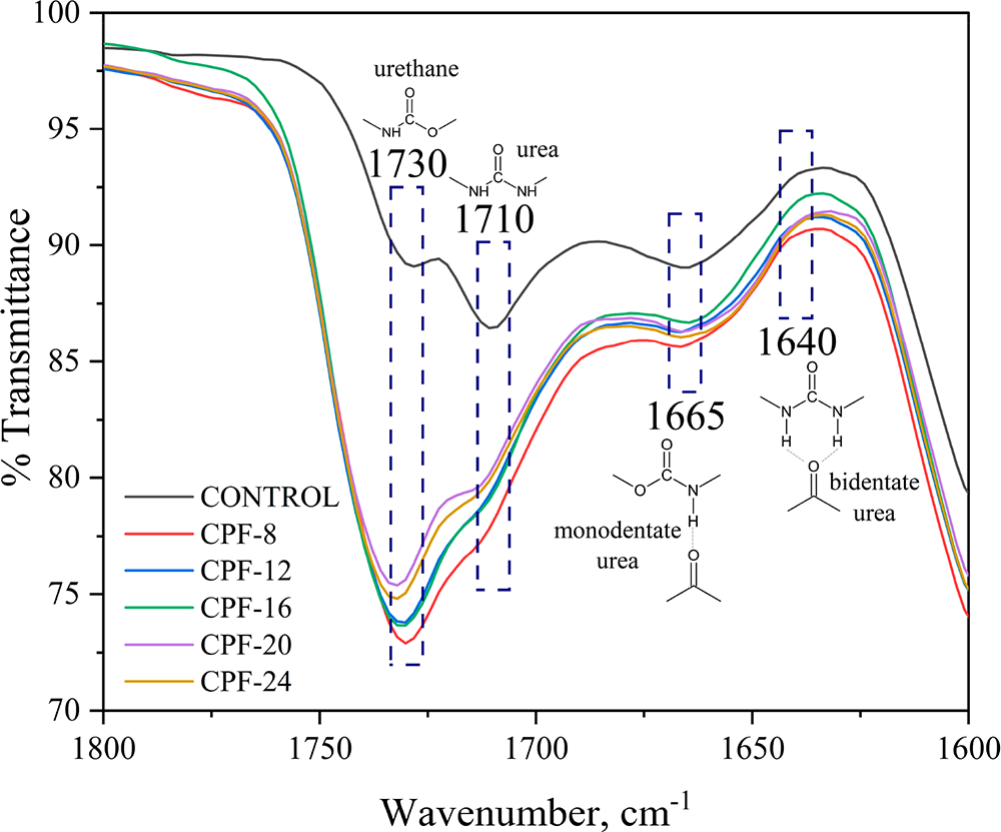
IR spectra of the CMGPOL-modified polyurethane foams (CPFs) and petroleum-based control foam highlighting the urethane–urea regions.

The effects of the incorporation of CMGPOLs in the structural characteristics of the foams are studied further by looking into the urethane–urea formation during the foaming process. Urethane is the primary product in the reaction between polyol and isocyanate, while urea arises from the blowing reaction between isocyanate and water. Urea formation is of particular importance as it has a crucial role in the cell structure and phase separation morphology of the polymer matrix.^55^ Moreover, the hydrogen bonding content of the urethane and urea needs to be inspected as it directly affects the mechanical properties of the foam.^51^ These groups and their corresponding IR peaks are indicated in Figure 10. Aside from the C=O stretching vibrations attributed to the esters in the CMGPOL, the band at 1727 cm^–1^ also corresponds to the C=O of urethanes. Another C=O stretching vibration appearing at 1710, 1665, and 1640 cm^–1^ signifies the presence of urea bonds and monodentate and bidentate H-bonded urea groups, respectively.^56^ The mentioned IR bands have greater absorbance intensities in the CPFs than in the control. This means that the formation of aggregated hard domains promoted by both monodentate and bidentate urea is predicted to be more pronounced in the CPFs than in the control. Aggregation of hard domains results in microphase separation,^57−60^ which further results in improved mechanical properties and increased cell opening.^55^ The foam samples in Figure 10 are arranged according to the highest monodentate and bidentate urea absorbance peaks. The following results showed similar trends in terms of the urea groups with almost the same behavior between CPF-24, -20, and -12:

- Monodentate urea: CPF-8 > CPF-24 > (CPF-20, CPF-12) > CPF-16 > CONTROL
- Bidentate urea: CPF-8 > (CPF-20, CPF-24, CPF-12) > CPF-16 > CONTROL

**Figure 11:**
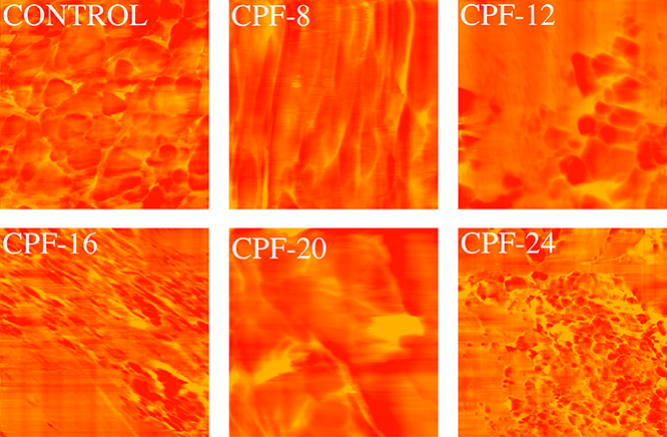
Atomic force microscopy (AFM) phase images of CMGPOL-modified polyurethane foams (CPF) and control foam measured with a size scan of 3 μm × 3 μm showing soft and hard regions represented by red and yellow colors, respectively.

Investigations using an atomic force microscope (AFM) enable the examination microphase separation and hard–soft phase distribution variations in the foam structure. This is done to confirm the observations derived from the IR spectra of the foams. The phase images of the foams measured at a scan size of 3 μm × 3 μm are depicted in Figure 11. It is evident in Figure 11 that the foam samples exhibit differing hard–soft distributions as there are separate segments characterized by different colors that represent varying degrees of modulus. The lighter color in the AFM images corresponds to the microdomains with higher modulus, relatively rigid, and isocyanate-rich regions. The darker regions denote the soft domains or the polyol-rich regions.

The foam samples in Figure 11 that exhibited a relatively high degree of microphase separation compared with other samples are CPF-8 and CPF-20. These foams appear to have relatively lighter areas of urea-rich regions separated more prominently from the darker, polyol-rich regions. In contrast, the control foam and CPF-16 show more dispersed hard and soft domains. CPF-24 and CPF-12 are at the middle of the scale, displaying light regions but with more dispersion than CPF-8 and CPF-20. These observations from the phase images of the foam samples are in agreement with the monodentate and bidentate urea contents of the samples, wherein the foams that exhibit greater H-bonding also manifest a higher degree of microphase separation. The same results were obtained by Baghban et al.^56^

**Figure 12:**
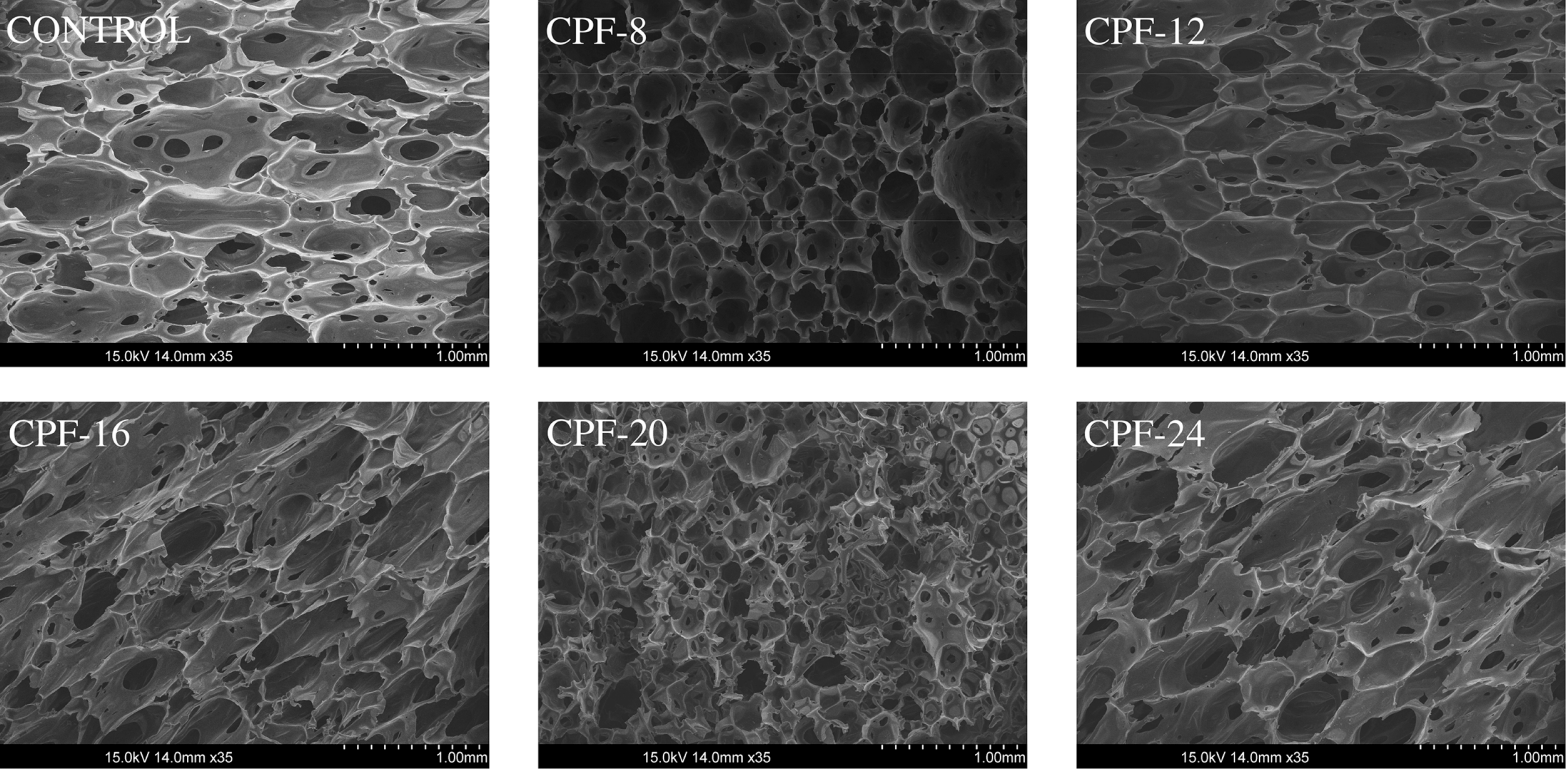
CMGPOL-modified polyurethane foams (CPFs) and petroleum-based control foam morphologies captured using a scanning electron microscope (SEM) at 35× magnification.

**Figure 13:**
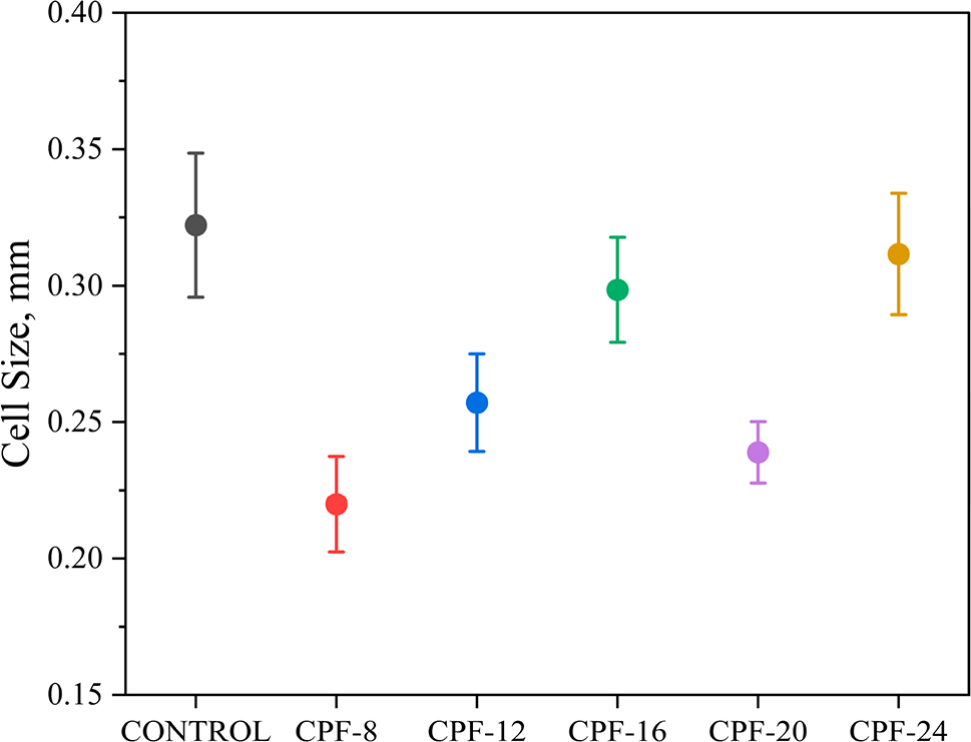
Cell size distribution of CMGPOL-modified polyurethane foams (CPFs) and petroleum-based control foam.

The foam morphologies were also examined using scanning electron microscopy (SEM), shown in Figure 12. Incorporating different CMGPOLs in the foam formulation significantly altered their cell sizes compared with the control. The CPFs have smaller cell sizes than the control, with CPF-8 having the smallest cell sizes. This contrast is quantified in Figure 13 where the cell size distribution of the foams is depicted. Aside from smaller cell sizes, the CPFs have a relatively more uniform cell size distribution. CPF-20 has the most uniform cell size among the foam samples. However, a significant degree of cell strut rupture can also be observed in Figure 12, specifically with CPF-16, -20, and -24.

Moreover, evaluation of the cell structures of the foam samples reveals a highly open-celled matrix. This finding is supported by the quantitative results from the determination of cell type content of the foams summarized in Table 6. All the CPFs recorded a slight increase in their open cell content compared with the control. These results can be explained by the aggregation of the hard segments due to strong H-bonding that induced more cell opening.^55^ This is consistent with the observed increase in the phase separation between hard and soft segments of the CPFs.

**Table 6:** Open Cell Content of CMGPOL-Modified Polyurethane Foams (CPFs) and Petroleum-Based Control Foam

foam samples	open cell content (%)
CONTROL	93.10 ± 0.21
CPF-8	96.13 ± 0.04
CPF-12	95.94 ± 0.32
CPF-16	94.80 ± 0.09
CPF-20	94.77 ± 0.80
CPF-24	95.42 ± 0.44

**Figure 14:**
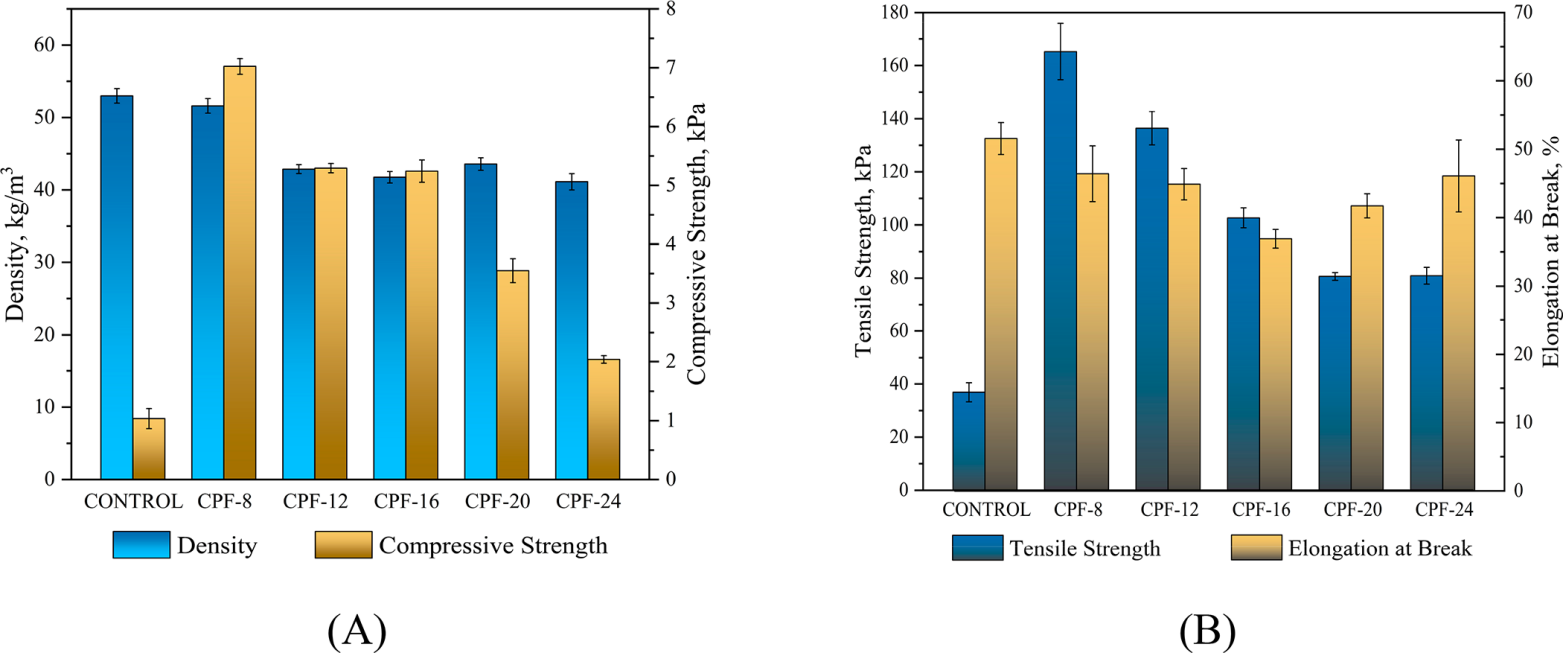
(A) Density and compressive force deflection at 50% deformation, and (B) tensile strength and elongation at break of CMGPOL-modified polyurethane foams (CPFs) in contrast with petroleum-based control foam.

Density is a vital property of PU foam as it is directly associated with its mechanical properties. The incorporation of CMGPOLs in the foam formulation decreased density, as depicted in Figure 14(A). CPF-8 only exhibited a slight decrease in density compared with the control, while the density of the rest of the CPFs significantly decreased. This behavior is likewise observed by similar studies, wherein a replacement of petroleum-based polyol with a biobased polyol at ≤20% led to a decrease in density.^9,61^ One factor that affected the density of the CPFs was the dangling chains on the backbone of CMGPOLs that rendered a plasticizing effect, thus lowering the density of the resulting foam.^62,63^ It can also be inferred that the higher OH content of the CMGPOLs led to higher NCO requirements. However, less reactive secondary OH groups are more predominant in the CMGPOLs, as indicated in “IR Spectra of Polyols” section. Thus, the NCO groups are more likely to favor the blowing reaction than the gelling reaction, producing more urea, as shown in Figure 10. According to Prociak and Rojek, the formation of more urea groups lowers the density of the foam.^9^

The compressive behavior of the CPFs in terms of CFD at 50% deformation is illustrated in Figure 14(A). The CPFs showed a notable increase in compressive strength compared with the control foam. This is attributed to the overall improvement in the mechanical behavior of the modified foams due to the higher OH number of the CMGPOLs that increased the hard segments in the foam matrix. In addition, the higher degree of phase separation, as indicated in Figure 11, has also contributed to the increase in compressive strength. This coincides with the findings of Abdollahi Baghban et al. and other related studies stating that strong, cohesive forces due to H-bonding have reinforced the hard segment linkages in the foam structure.^56,64,65^

Parallel to the compressive properties of the CPFs in Figure 14(A), their tensile strength showed similar behavior, as depicted in Figure 14(B). All the CPFs displayed a striking increase in their tensile strength compared with the control, despite the slight reduction in density. CPF-8 exhibited the highest increase at ca. 350%. This improvement in the tensile capacity of the foams can be ascribed to the higher number of hard segments in the CPFs than the control, shown in Figure 10, owing to the higher OH groups in CMGPOLs. However, Figure 14(B) reveals a slight decrease in the elongation at break of the CPFs than the control. This general decrease in elongation at break is owed to the lower molecular weight of the CMGPOLs incorporated in the foam formulation than the pure petroleum-based polyol used in the control foam, which resulted in shorter soft segment lengths.^66^ It can be observed that CPF-16 has the most sizable decrease in elongation at break. This can be explained by the more ruptured and bigger cell sizes of CPF-16 compared with the other foams, as shown in Figure 12 which led to a decrease in elongation at break.^67,68^

The phase separation of the hard and soft segments in the foam matrix brought by the increased H-bonding of urea groups has also been found to influence the resilience of flexible PU foam. Table 7 lists the resilience of the foam samples determined using the ball rebound test. An overall increase in the resilience performance of the CPFs can be observed when compared with the control, except for CPF-16, where resilience slightly decreased. This outcome appears to have an inverse relationship with phase separation. Among the CPFs, CPF-16 has the least degree of H-bonded urea that promoted phase separation, as shown in Figure 10. In contrast, CPF-8 and -24, arguably having the highest degree of phase separation, showed the highest increase in resilience. This derived inference is in accordance with the findings of similar studies that inversely correlated the resilience of PU foams to the degree of hard and soft segment phase separation.^69,70^

**Table 7:** Resilience of the CMGPOL-Modified Polyurethane Foams (CPFs) in Comparison with Petroleum-Based Control Foam

foam sample	resilience (%)
CONTROL	16 ± 2
CPF-8	28 ± 3
CPF-12	23 ± 1
CPF-16	13 ± 2
CPF-20	22 ± 2
CPF-24	30 ± 3

**Figure 15:**
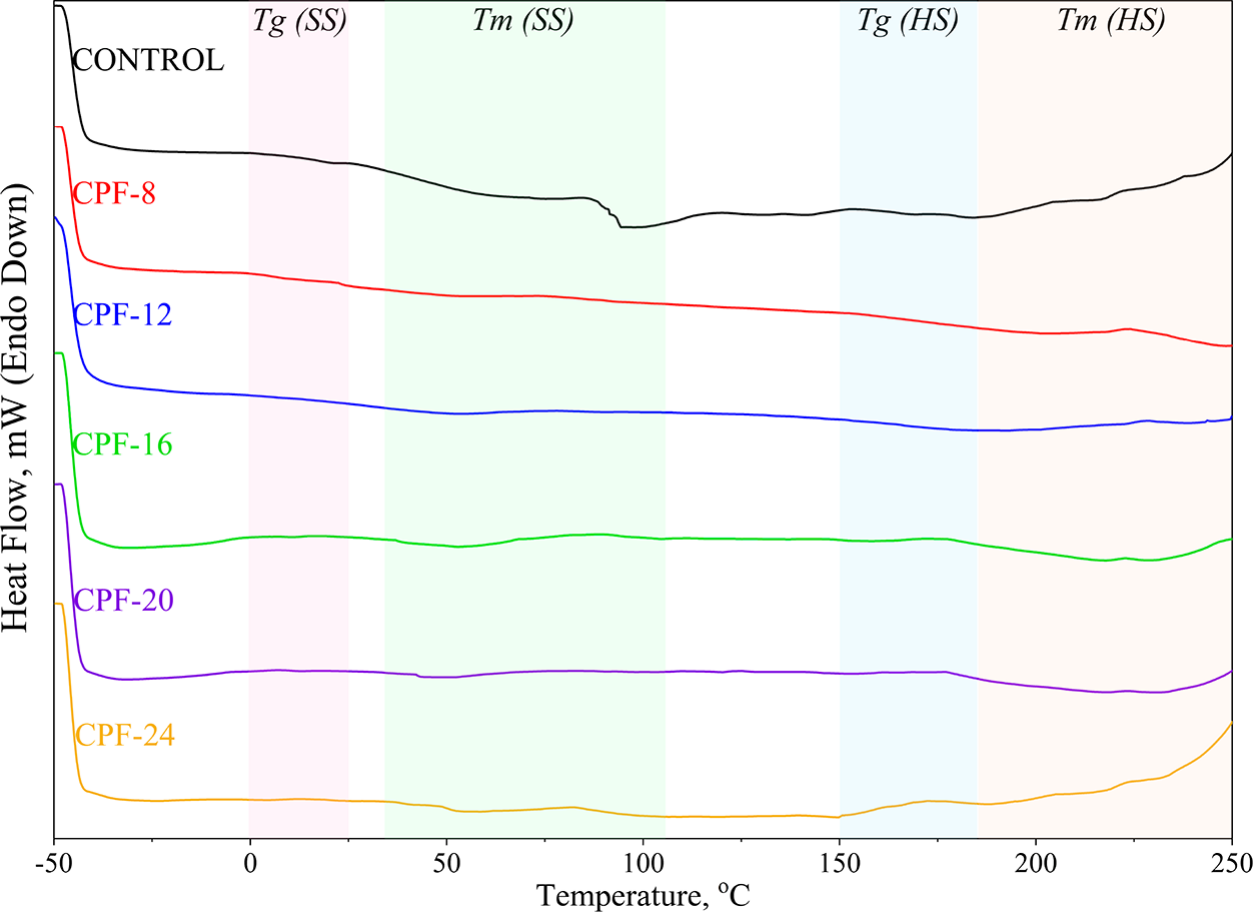
Differential scanning calorimetry (DSC) of different CMGPOL-modified polyurethane foams (CPF) along with petroleum-based control foam showing the glass transition temperatures (*T*_g_) and melting temperatures (*T*_m_) of soft segments (SS) and hard segments (HS).

The thermal profiles of the foams were examined using DSC. Due to microphase separation rendering some degree of thermodynamic incompatibility between the hard and soft segments, four distinct thermal properties of the foams were detected: the glass transition temperatures (*T*_g_) and melting temperatures (*T*_m_) of the soft segments (SS) and hard segments (HS).^71^ These regions are depicted in Figure 15 and the results are elaborated in Table 8.

**Table 8:** Summary of the Glass Transition Temperatures (*T*_g_) and Melting Temperatures (*T*_m_) of Soft Segments (SS) and Hard Segments (HS) of the Different CMGPOL-Modified Polyurethane Foams (CPF) along with Petroleum-Based Control Foam

	soft segments (SS)		hard segments (HS)	
sample	*T*_g_, °C	*T*_m_, °C	*T*_g_, °C	*T*_m_, °C
CONTROL	12.4	93.5	161.6	183.7
CPF-8	16.4	52.4	185.1	223.2
CPF-12	11.9	53.0	177.7	201.6
CPF-16	9.4	53.6	162.6	217.2
CPF-20	9.9	51.5	171.2	217.7
CPF-24	1.9	55.5	174.1	208.2

According to Table 8, the *T*_g_ (SS) of the CPFs falls within the range of 1.9–16.4 °C, while the control has a *T*_g_ (SS) of 12.4 °C. A decreasing shift in *T*_g_ (SS) can be observed among the CPF samples as CMG loading increases, with CPF-8 having the highest *T*_g_ (SS). Similarly, CPF-8 has the highest *T*_g_ (HS) of 185.1 °C, while the other foam samples fall only between 161.6 and 177.7 °C. This behavior may be ascribed to CPF-8 appearing to have the highest degree of phase separation, thus shifting its *T*_g_ (SS) to higher temperatures.^72^

Additionally, the *T*_m_ (SS) of the samples are also outlined in Table 8. The CPFs have almost similar melting peaks between 51.5 and 55.5 °C. However, a different behavior can be observed with the melting peak of the control sample at 93.5 °C. This is in contrast with the *T*_m_ (HS) of the control foam, having the lowest value of 183.7 °C, while CPF-8 has the highest value of 223.2 °C. The dissimilitude in the *T*_m_ of the SS and HS of the control sample can be attributed to the greater ordered structure in the SS of the PU matrix due to a more uniformed structure of the petroleum-based polyol, as opposed to the branched structure of CMGPOL with its dangling chains.^73,74^ Furthermore, the high *T*_m_ (HS) of the CPFs verifies the presence of higher HS content compared with the control. The highest recorded *T*_m_ (HS) of 223.2 °C for CPF-8 coupled with its *T*_g_ (HS) of 185.1 °C verifies the high degree of phase separation in this sample.

**Figure 16:**
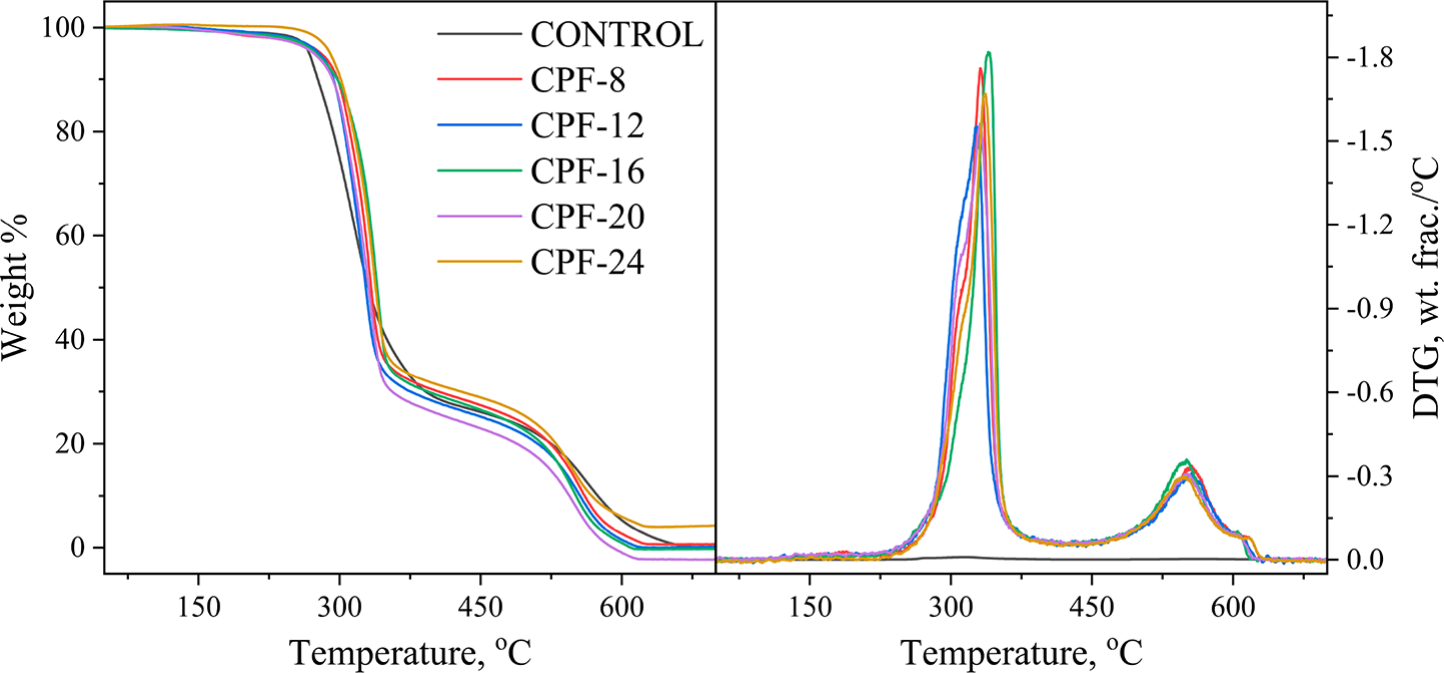
TG and DTG curves of different CMGPOL-modified polyurethane foams (CPF) and petroleum-based control foam.

The TG and DTG curves of the flexible foams are displayed in Figure 16, and the details of thermal degradation are summarized in Table 9. The foams exhibited almost similar decomposition behavior, as shown in Figure 16. The slight weight loss (<5%) between 100 and 200 °C may be attributed to moisture evaporation in the samples. It can be discerned that the foam samples have three distinct degradation peaks. The degradation profile of the samples can be understood more clearly by examining the degradation peaks and extent of sample weight loss, as tabulated in Table 9. The first degradation peak, T_1_, corresponds to the degradation of the urethane bonds evolving polyol and isocyanate. This coincides with the findings of Gu and Sain that the decomposition peak of the urethane bonds in flexible foams can be found between 230 and 380 °C under air.^75^ It can be observed that the T_1_ of the CPFs is higher than the control. These results denote improvement in the thermal stability of the foam with the CMGPOL content owing to the higher OH group content of CMGPOL that increased the density of urethane linkages. The second degradation peak, T_2_, accounts for the thermo-oxidative degradation of soft segments, while the third peak, T_3_, refers to the decomposition of isocyanate. It is important to note that T_2_ and T_3_ of the CPFs are slightly lower than the control. This may be ascribed to the mutual stabilizing effect of the hard and soft segments in the CPFs, wherein thermal stability increases in the initial stage of degradation, while the opposite is true at later stages.^76^

**Table 9:** Thermal Degradation Temperatures and Weight Loss of CMGPOL-Modified Polyurethane Foams (CPF) and Petroleum-Based Control Foam

sample	*T*_max_, °C			% weight loss		
	T_1_	T_2_	T_3_	m_1_	m_2_	m_3_
CONTROL	312.8	562.5	630.3	72.3	26.6	1.0
CPF-8	331.3	555.8	617.0	69.5	24.9	2.1
CPF-12	328.4	556.3	609.1	73.6	24.4	1.4
CPF-16	339.3	549.7	606.4	70.5	27.2	0.3
CPF-20	331.0	550.9	604.5	74.5	25.0	0.1
CPF-24	336.4	547.4	617.2	69.0	24.6	1.9

### Implications to Sustainability in the PU Industry

In line with the tripartite concept of sustainability encompassing the economic, social, and environmental aspects,^77^ sustainability in industrial development relies heavily on the utilization of feasible technologies that have beneficial socio-economic and environmental impacts.^78^ The use of renewable resources bears significant weight in the overall sustainability of an industry as this is directly linked to the human quality of life. Excessive resource consumption correlates to the threat of diminishing quality of life, and economic growth increases this threat.^79^ With the continued economic upturn of the PU industry expanding its influence to a vast network of different industries,^10^ the choice of raw materials is crucial. Although existing and emerging technologies offer biobased materials as alternative feedstocks, petroleum-based materials still dominate the PU industry, exacerbating the threat to humans and the environment as the latter is among the top emitters of greenhouse gases.^80^ Also, the economic instability of the petroleum industry due to its highly volatile prices and increasing global demand can negatively impact the economics of the PU industry. Thus, the preparation of polyols from coconut oil-derived materials, such as CMG, offers another step toward curtailing fossil oil dependency and boosting the sustainable polymer market.

However, the use of biobased materials in polyol and PU foam synthesis, despite adhering more to green design than petroleum-based materials, can still have negative ramifications. Problems such as augmenting the pressure on the food economy, health hazards, and environmental pollution may arise.^81^ In this work, the raw material, CMG, is a coproduct of fatty acid production,^21^ which is classified as a second-generation feedstock and does not directly intrude on the food industry.^82^ As for the environmental and health hazards in biobased polyol synthesis, one major contributing factor is the use of dangerous and toxic chemicals as solvents and catalysts.^3,8,9,83^ Exposure to these chemicals can pose serious health issues, and their handling, use, and disposal can be avenues for environmental problems. However, this work produced a polyol product from CMG through a solvent-free and uncatalyzed polycondensation process with water as a byproduct. Hence, the employed process has purported lower environmental and health hazards than other processes, which can be evaluated via a full life cycle assessment (LCA).

## Conclusions

CMG-based polyols (CMGPOLs) with high-molecular-weight have been successfully synthesized. Assessment of the polyol properties showed their potential as precursors in flexible PU foam production. The number-average molecular weight of the polyols at 1997–4275 g/mol satisfies the requirements of flexible foam at an average molecular weight of 1000–6000 g/mol. The OH number of the polyols at 77–142 mg KOH/g falls within the target of 28–160 mg KOH/g, while their functionality is recorded at 4.8–5.8. Three out of five polyol products met the industrially acceptable acid number of <10 mg KOH/g and viscosity of <50 Pa·s for polyols. In terms of molecular structure, the fatty acid esters on CMG can act as an internal plasticizer, which may benefit flexible foams. The CMGPOLs also showed low *T*_g_ values and no crystallization owing to the dangling fatty acid chains speculated to impart hydrolytic stability on the polyols. TGA analysis showed high thermal degradation temperature of the products. In addition, evidence of the polyols’ potential application was demonstrated through the production of CMGPOL-modified PU foams (CPFs) at 20 wt % loading. Results showed ca. 120–350% increase in tensile strength, CFD of 2–7 kPa at 50% deformation, density of 41.13–51.61 kg/m^3^, open cell content of >94%, and resilience between 13–30%. SEM images of the CPFs showed a decrease in cell size and distribution. CPF-8 displayed the highest HS content and phase separation among the samples, verified by its high *T*_m_ and *T*_g_. The thermal decomposition profile of the CPFs was also investigated, revealing better thermal stability, especially at the initial stage of decomposition. These enhancements in the properties of the CPFs were attributed to the increase in monodentate and bidentate urea groups, analyzed using FTIR, that promoted microphase separation in the polymer matrix. This was confirmed by inspecting the phase images of the foams using AFM. This study not only successfully developed novel biobased polyols from CMG through a solvent-free and uncatalyzed polycondensation process and produce flexible foams with improved properties but also opened many possibilities for future work.

## Abbreviations

FPUF: flexible polyurethane foam
CMG: coconut monoglycerides
CMGPOL: CMG-based polyester polyol
CPF: CMGPOL-modified PU foam
